# Impact of plant genotype and plant habitat in shaping bacterial pathobiome: a comparative study in olive tree

**DOI:** 10.1038/s41598-020-60596-0

**Published:** 2020-02-26

**Authors:** Diogo Mina, José Alberto Pereira, Teresa Lino-Neto, Paula Baptista

**Affiliations:** 1 Centro de Investigação de Montanha (CIMO), Instituto Politécnico de Bragança, Campus de Santa Apolónia, 5300-253 Bragança, Portugal; 2 Biosystems & Integrative Sciences Institute (BioISI), Plant Functional Biology Center (CBFP), University of Minho, Campus de Gualtar, 4710-057 Braga, Portugal

**Keywords:** Bacteria, Microbial communities

## Abstract

Plant-inhabiting microorganisms interact directly with each other affecting disease progression. However, the role of host plant and plant habitat in shaping pathobiome composition and their implication for host susceptibility/resistance to a particular disease are currently unknown. For the elucidation of these questions, both epiphytic and endophytic bacterial communities, present in asymptomatic and symptomatic twigs from olive cultivars displaying different susceptibilities to olive knot (OK) disease, were investigated using culturing methods. OK disease was the main driver of the bacterial community, causing changes on their diversity, abundance and composition. OK disease effect was most notorious on OK-susceptible cultivar and when considering the endophytic communities. Plant habitat (epiphytes *vs*. endophytes) also contributed to the bacterial community assembling, in particular on symptomatic twigs (knots) of OK-susceptible cultivar. In contrast, host cultivar had little effect on the bacterial community composition, but OK-symptomatic twigs (knots) revealed to be more affected by this driver. Overall, the pathobiome seems to result from an intricate interaction between the pathogen, the resident bacteria, and the plant host. Specific bacterial genera were associated to the presence or absence of OK disease in each cultivar. Their ability to trigger and/or suppress disease should be studied in the future.

## Introduction

It is now well established that plants harbor a complex microbial community (microbiota) that provides numerous health benefits^1^. From the various mechanisms employed by microbes to improve host plant health, microbe-microbe interactions seem to play fundamental roles^2^. Indeed, there are some studies indicating that within plant microbiota, pathogens can establish multiple interactions, either positive or negative, with other microorganisms that may trigger or influence the disease process^3,4^. Such microbial consortium, which play a direct role on the progression of disease, has been recently termed as pathobiome^3^. Although in this concept the pathogenic agent has been regarded as integrated within this biotic environment^3^, the host plant role in shaping the pathobiome and its implication for host susceptibility/resistance to a particular disease have not yet been studied. As the structure of plant-associated microbiota is plant genotype dependent^5,6^, we hypothesized that distinct microbial compositions among plant genotypes with diverse microbial interactions may lead to different pathobiomes. Another critical question is whether the pathobiome composition depends on plant habitat. Plant-associated microorganisms have the ability to colonize the surface (epiphytic) or the internal (endophytic) plant tissues^1^. However, whether the microbial interactions in the plant tissue surface may lead to different pathobiomes than those from the interior of the same plant tissues is still largely unknown. Microbiota comparisons (either epiphytic or endophytic) between healthy and diseased plant tissues, present in cultivars with contrasting susceptibility to diseases, could be helpful to elucidate these questions^7^. Such approach, besides providing new insights on the potential role of microbiota in plant resistance, could additionally contribute for the identification of microbial strains that could be used in the future as “probiotic”. The application of such probiotic microorganisms could drive the plant microbiota towards a pathogen-resistant microbial composition. In humans for instance, the faecal microbial transplantation has been largely recognized as a promising therapy to treat gastrointestinal diseases^8^.

*Pseudomonas savastanoi* pv*. savastanoi* (*Pss*) is the causal agent of the olive knot (OK) disease, which is one of the major threats to olive tree (*Olea europaea* L.) production in most olive growing regions of the world, in particular Mediterranean region^9,10^. *Pss* lives epiphytically on the surface of olive organs^9^, and under favorable weather conditions, *Pss* population increase and penetrate into olive tissues, leading to the formation of tumorous overgrowths^10^. These knots are deeply colonized by *Pss* microcolonies and comprise the main symptoms of OK disease, occurring mostly on olive tree twigs, branches and trunks^11^. Several non-pathogenic bacterial species from these knots have been reported to cooperate with the *Pss* for increasing disease severity^12–14^. Therefore, these knots can provide an excellent model system for studying the impact of plant host and plant habitat on the pathobiome structure. So far, no olive tree genotype has been found to be completely resistant to OK, but different olive cultivars exhibit different susceptibilities to OK disease^15^. For instances, among the most important Portuguese commercial olive cultivars, cv*. Cobrançosa* is less susceptible to OK than cv. *Verdeal Transmontana*^16^. Both cultivars can simultaneously display asymptomatic twigs and symptomatic twigs (with knots) in the same olive tree, also making this system suitable for studying the impact of host genotype on the “health” *vs*. “disease” (*i.e*., pathobiome) microbiota structure.

Here, we investigated the epiphytic and endophytic bacterial community of asymptomatic and symptomatic (knots) twigs, present in olive cultivars of different susceptibilities to OK (cvs. *Cobrançosa* and *Verdeal Transmontana*), using culturing methods followed by sequencing of the PCR amplicons of bacterial isolates. Such approach would allow to capture the complex pathogen-microbe-plant interactions and predict links between the cultured microbial community and disease/healthy states. The possible contribution of such links for the different susceptibilities of cultivars to OK disease could also be elucidated. Therefore, with this work we aim to answer the following questions: (i) May host cultivar shape the associated pathobiome community? (ii) Is the pathobiome composition dependent on the plant habitat (epiphytic *vs*. endophytic)? (iii) Is there any bacterial consortium specifically associated to asymptomatic (“healthy-promoting microbiota”) twigs or to knots (“disease-promoting microbiota”)? (iv) Are “healthy- or disease-promoting microbiota” linked to cultivar susceptibility to OK disease? The isolation of bacteria with a potential role on plant resistance to OK disease could be a first step for envisaging a biocontrol strategy for the inhibition of olive knot disease.

## Results

The isolation of bacterial epiphytes and endophytes from asymptomatic and OK-symptomatic (knots) twigs from the 28 olive trees of both cultivars (*Cobrançosa* and *Verdeal Transmontana*) resulted in a total of 312 isolates. All isolates, corresponding to 66 operational taxonomic units (OTUs), belong to 31 genera and 17 families, mostly from the *Proteobacteria* and *Actinobacteria* phyla (76.3% and 18.2% of the total bacterial isolates, respectively) (Fig. S1). Considering all OTUs, 68.2% were found on the surface and 56.1% in the interior of plant tissues. The epiphytic bacterial communities were predominantly dominated by members belonging to genera *Pseudomonas* and *Curtobacterium* accounting together for 83.2% of total epiphytes, whereas *Pseudomonas* and *Pantoea* were dominant in the endophytic community, accounting together for 71.3% of the total endophytic isolates. Although all surveyed environments were colonized by *Pss*, the pathogen abundance was significantly higher in knots (OK-symptomatic twigs) than in asymptomatic twigs, either for epiphytic (2.0-fold, *p *< 0.001) or endophytic (2.1-fold, *p *< 0.001) communities (Table S1). Curiously, the more resistant cv. *Cobrançosa* presented higher abundance of *Pss* than the OK-susceptible cv. *Verdeal Transmontana* (1.9-fold, *p *< 0.001) within epiphytic community, but within the endophytic community was observed the opposite (a reduction of 1.4-fold, *p *< 0.001).

### Comparison of bacterial communities

**Figure 1:**
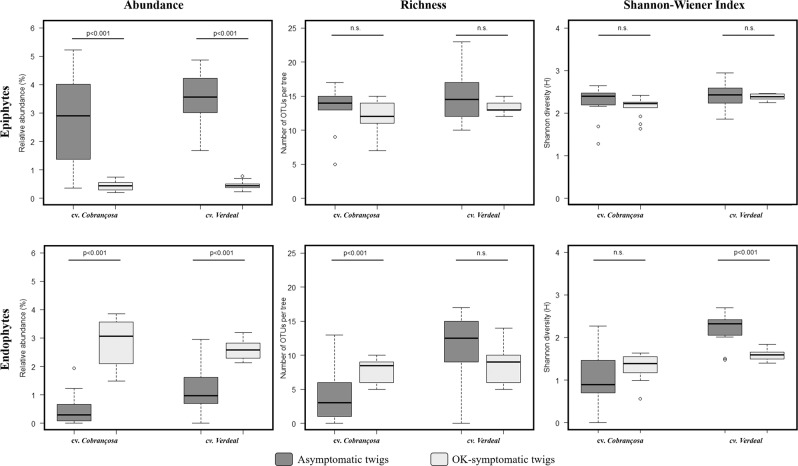
Comparison of bacterial diversity between asymptomatic and OK-symptomatic (knots) twigs, either within endophytic or epiphytic communities from each olive tree cultivar (*Cobrançosa* and *Verdeal Transmontana*). Diversity at community level was evaluated by determining bacterial abundance, richness and by using Shannon–Wiener index. Box plots depict medians (central horizontal lines), the inter-quartile ranges (boxes), 95% confidence intervals (whiskers), and outliers (black dots). Significant differences between pairs of values are represented over horizontal lines.

The abundance and diversity of bacteria differed between asymptomatic and OK-symptomatic (knots) twigs, depending also on the host cultivar and plant habitat (Figs. 1 and S2). Although a significant reduction (*p *< 0.001) in epiphytes abundance was detected from asymptomatic to OK-symptomatic twigs (up to 6.9-fold), an opposite result was observed for endophytes (an increase of 3.2-fold). This increase on endophytic abundance was significantly (*p *< 0.05) greater on cv. *Cobrançosa* (83.3%) when compared to cv. *Verdeal Transmontana* (52.9%). The bacterial diversity (determined by the species richness and Shannon-Wiener diversity index) was only significantly different between asymptomatic and OK-symptomatic twigs for the endophytic community. In cv. *Cobrançosa*, the richness of endophytes increased significantly (up to 2.2-fold, *p *< 0.001) from asymptomatic to OK-symptomatic twigs, whereas an opposite result was observed in cv. *Verdeal Transmontana* when considering Shannon-Wiener diversity index (a decrease up to 1.4-fold, *p *< 0.001).

**Figure 2:**
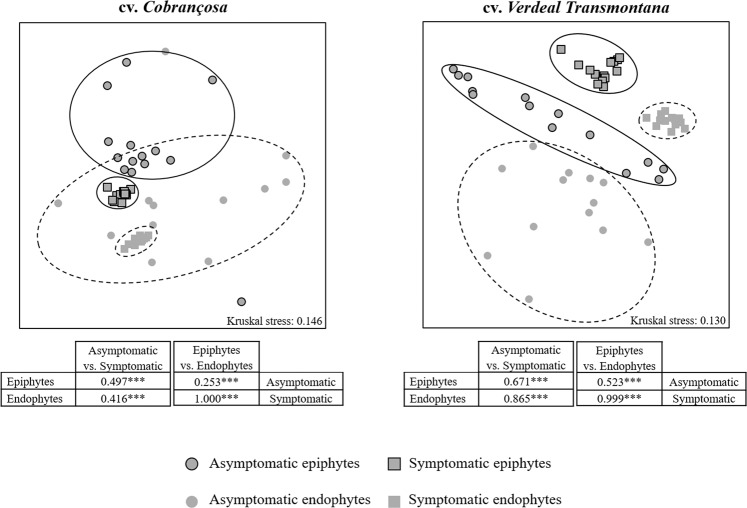
Nonmetric multidimensional scaling (NMDS) plots and ANOSIM tests for the bacterial assemblages in twigs of olive trees from cvs. *Cobrançosa* and *Verdeal Transmontana*, considering the presence of OK disease symptoms (asymptomatic *vs*. OK-symptomatic) and plant habitat (epiphytic *vs*. endophytic). Bray-Curtis coefficient was used as a measure of similarity between populations and Kruskal’s stress values are presented (values less than 0.2 represent good ordination plots). ANOSIM test showed the *R*-statistics (*R*) and the statistical significance, which is denoted by asterisks (*p < 0.05; **p < 0.01; ***p < 0.001).

The whole bacterial community composition significantly differs between asymptomatic and OK-symptomatic (knots) twigs, as revealed by the non-metric multidimensional scaling (NMDS) plots and analysis of similarities (ANOSIM; *R* = 0.255, *p *< 0.001) based on Bray-Curtis index (Fig. 2). These differences were higher on the OK-susceptible cv. *Verdeal Transmontana*, either within epiphytic (*R* = 0.671, *p *< 0.001) or endophytic (*R* = 0.865, *p *< 0.001) communities, than in the more resistant cv. *Cobrançosa* (*R* = 0.497 and *R* = 0.416 with *p *< 0.001, respectively). In addition, the dissimilarity found on bacterial composition between epiphytic and endophytic communities was always greater in OK-symptomatic twigs (*R* = 1.000 and *R* = 0.999 with *p *< 0.001, for cv. *Cobrançosa* and *Verdeal Transmontana*, respectively) than in asymptomatic twigs (*R* = 0.253 and *R* = 0.523 with *p *< 0.001, respectively).

**Figure 3:**
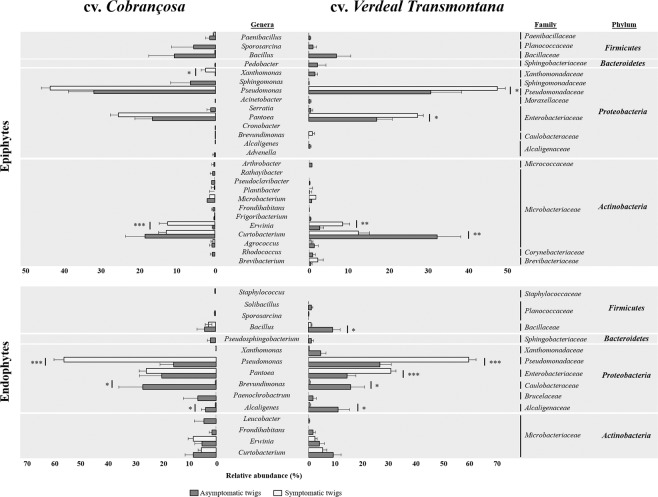
Comparison of the relative abundance of bacterial epiphytes and endophytes, between asymptomatic and OK-symptomatic twigs of olive trees from cvs. *Cobrançosa* and *Verdeal Transmontana*. Comparisons are made considering different taxonomic levels (genus, family and phylum). Each value is expressed as mean ± standard error (n = 14, corresponding to 2 olive orchards × 7 trees). Statistically differences between pairs of values are showed by asterisks (*p < 0.05; **p < 0.01; ***p < 0.001).

The taxonomic differences of epiphytes and endophytes in asymptomatic and OK-symptomatic twigs were evaluated by comparing differences on the relative abundances at genus, family and phylum levels (Fig. 3). Overall, both asymptomatic and OK-symptomatic twigs (from both olive cultivars) were dominated by bacterial isolates belonging to *Pseudomonadaceae* family (*Proteobacteria* phylum), accounting for 26.8% and 51.7% of the total number of isolates obtained in each twig type, respectively. *Microbacteriaceae* and *Enterobacteriaceae* were the second most representative families of asymptomatic and OK-symptomatic twigs, representing together 24.4% and 27.3% of the total isolates in each sample type, respectively. As previously revealed, the bacterial communities of each cultivar were differently affected by OK disease. In cv. *Cobrançosa*, a significant increase in *Xanthomonas* (up to 106.4-fold), *Erwinia* (25.5-fold) and *Pseudomonas* (3.6-fold), as well as a significant decrease on *Brevundimonas* (140.0-fold) and *Alcaligenes* (16.4-fold) were observed in OK-symptomatic twigs (knots) in relation to asymptomatic twigs. In contrast, slighter changes occurred in cv. *Verdeal Transmontana*, where minor (but significant) increases were detected in the abundance of *Erwinia* (up to 3.2-fold), *Pseudomonas* (up to 1.5-fold) and *Pantoea* (up to 1.6-fold), as well as a significant decrease on *Curtobacterium* (up to 2.6-fold). However, the number of bacterial genera that disappeared with OK disease was greater in cv. *Verdeal Transmontana* (in total 19) when compared to cv. *Cobrançosa* (in total 15). Furthermore, in the OK-susceptible cultivar (cv. *Verdeal Transmontana*), *Bacillus* and *Alcaligenes* genera were only present on asymptomatic twigs, while *Brevundimonas* was only isolated from OK-symptomatic tissues.

### Contribution of different drivers for bacterial community shaping

For determining the relative contribution of host cultivar, occurrence of OK-symptoms and plant habitat (epi- or endophytic) in shaping the bacterial community, a variation partitioning analysis was performed (Table S2). Results revealed that bacterial composition in twigs was mainly explained by the absence/presence of OK-symptoms (responsible for 7.3% of the total variation) and plant habitat (epi- or endophytic, 7.1% of the total variation), contrasting with host cultivar that only explained 3.6% of the total community variation. The amount of variance explained by the occurrence of OK-symptoms was greater in cv. *Verdeal Transmontana* (20.5%) and endophytic (11.7%) communities. Plant habitat mainly affected the bacterial composition in cv. *Verdeal Transmontana* and symptomatic twigs, explaining 13.8% and 26.8% of species composition variance, respectively. Host cultivar had a higher influence on OK-symptomatic (24.4%) twigs and epiphytic (14.7%) communities.

### Potential bacteria consortia for olive tree susceptibility/resistance

**Figure 4:**
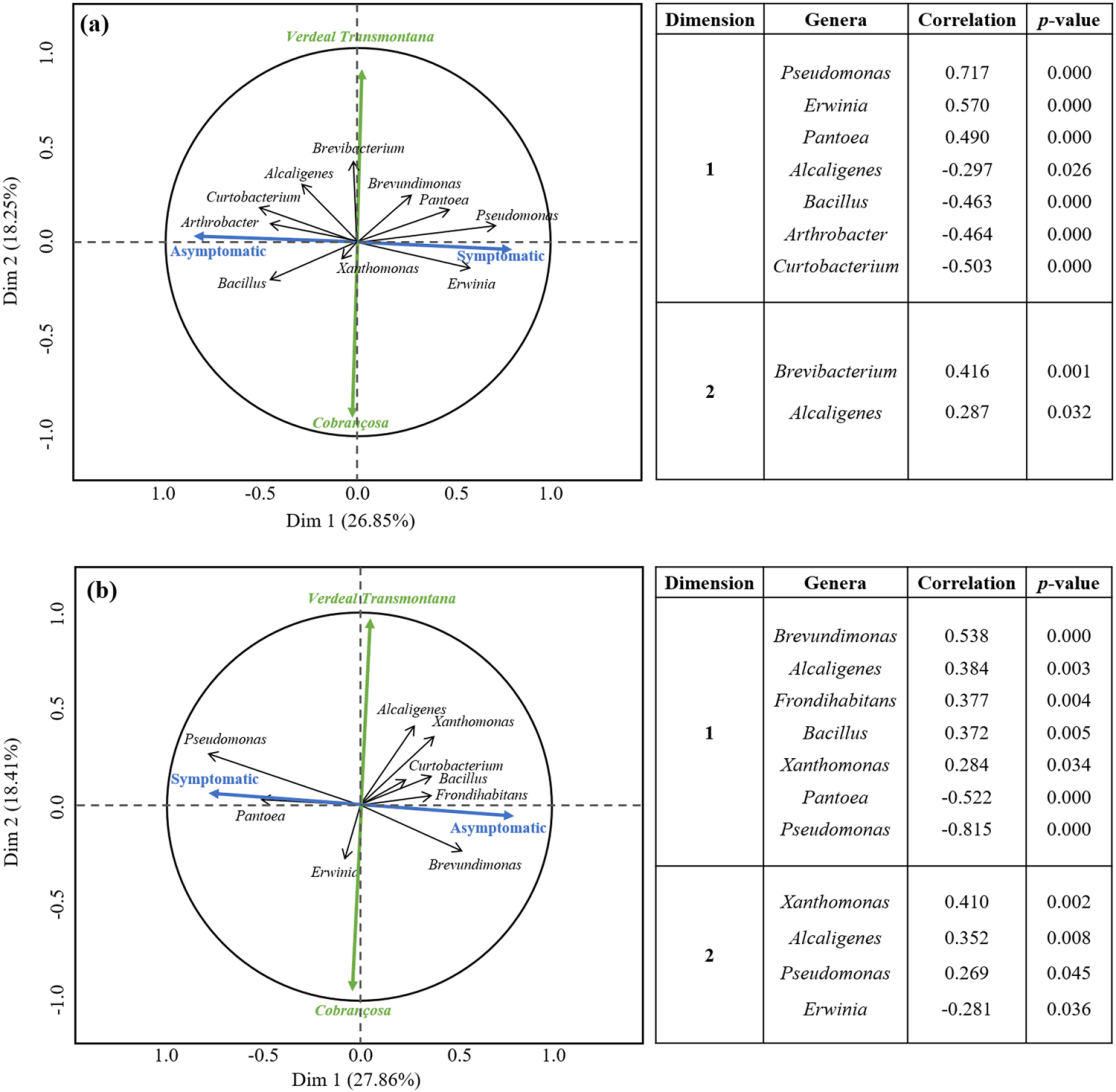
Circle plot of multiple factor analysis (MFA) correlations of bacterial abundance among olive cultivars (*Cobrançosa* and *Verdeal Transmontana*) and the presence of OK disease symptoms (asymptomatic and OK-symptomatic), when applied to epiphytic (**a**) and endophytic (**b**) bacterial communities. On the right, the correlation table between each dimension and bacteria genera is shown.

One goal of this study was the identification of a set of bacterial genera associated to asymptomatic twigs or knots (OK-symptomatic twigs) and elucidate if these bacterial consortia could explain differences in OK disease susceptibility of olive tree cultivars. To more accurately predict such relationships, a random forest analysis was employed to rank the importance of bacterial genera in distinguishing either asymptomatic from OK-symptomatic twigs (Fig. S3) and cv. *Cobrançosa* from cv. *Verdeal Transmontana* (Fig. S4). According to their Gini coefficient value (higher the value, greater its importance)^17^, ten and nine different bacterial genera were selected as the most important in discriminating twigs with/without OK disease symptoms and host cultivar, respectively (Figs. S3 and S4). These bacterial genera were used to perform a multiple factor analysis (MFA), in order to find relationships between bacterial genera and presence/absence of OK symptoms and/or susceptibility/resistance of cultivar to OK disease (Fig. 4). In this analysis, the first dimension revealed a clear opposition between bacteria present in asymptomatic and in knots (OK-symptomatic twigs), either within epiphytic (Fig. 4a) or endophytic (Fig. 4b) bacterial communities. *Pseudomonas*, *Erwinia* and *Pantoea* were positively correlated with the presence of OK disease in the epiphytic community, as well as in the endophytic community (except *Erwinia*). This result is corroborated by the significantly positive correlation of these genera with *Pss* abundance (Table S3). On the other hand, *Alcaligenes* and *Bacillus* were positively correlated with asymptomatic twigs, either in the epiphytic and endophytic communities. In addition, *Arthrobacter* and *Curtobacterium* (in epiphytic community) and *Brevundimonas*, *Frondihabitans*, and *Xanthomonas* (in endophytic community) were positively correlated with asymptomatic twigs. Some of these bacterial genera were also found to be negatively correlated with *Pss* abundance (*Bacillus*, *Curtobacterium*, and *Brevundimonas*; Table S3). The second dimension of the MFA ordination of both epiphytic and endophytic bacterial communities clearly separated both olive cultivars (Fig. 4). *Brevibacterium* and *Alcaligenes* in epiphytic community, as well as *Xanthomonas*, *Alcaligenes* and *Pseudomonas* in endophytic community, were positively correlated with cv. *Verdeal Transmontana*. *Erwinia* was the only genus found to be specifically associated to cv. *Cobrançosa*.

## Discussion

With this work, we attempted to disclose the role that host plant-microbe-pathogen interactions may have in the development of OK disease and reveal the underlying pathobiome. For this, epiphytic and endophytic bacterial communities from asymptomatic and OK-symptomatic olive twigs, taken from olive tree cultivars with contrasting susceptibilities to OK disease, were compared. Greater differences on bacterial abundance, diversity and composition were detected for the OK-susceptible cv. *Verdeal Transmontana* compared to OK-resistant cultivar. Thus, the olive cultivar seems to influence the establishment of pathobiome communities in olive knots. Accordingly, previous studies have suggested that differences on microbial abundance and diversity between asymptomatic and symptomatic tissues were possibly related to the susceptibility of the plant host to a certain disease^16,18–21^. We hypothesized that the detected greater differences in the bacterial composition of OK-susceptible cv. *Verdeal Transmontana*, when compared to cv. *Cobrançosa*, may be due to differential metabolite alterations occurring on both cultivars upon *Pss* infection. Indeed, plants can defend themselves against pathogens by a variety of mechanisms that enable the detection of pathogen invasion and activation of a defense response^22^. This defensive response is highly complex and involves a cellular reprogramming that is characterized by an altered plant metabolism with the biosynthesis of defensive compounds^23^. Although there are still gaps in understanding the dynamism and complexity of such metabolic alterations^23^, recent studies have indicated that this response is cultivar dependent^24^. Our hypothesis is further reinforced by the *Varpart* analysis, which showed a greater contribution of host cultivar to the bacterial assemblage in OK-symptomatic twigs (24.4%) when compared to asymptomatic twigs (2.5%), as similarly observed for the fungal community in the same olive tree cultivars^16^. The role of host plant in structuring both rhizosphere and root endosphere bacterial communities in response to a pathogen attack was already reported^25,26^. In these studies, plants subjected to pathogen attack were proposed to recruit protective bacteria for suppressing pathogens in the rhizosphere^25^. We hypothesize that the same could be occurring in the olive tree phyllosphere upon *Pss* infection. However, further studies should be conducted to confirm such effect in our pathosystem, as differences on bacterial composition between olive tree cultivars may also be due to *Pss* itself. For example, mammalian bacterial pathogens have been described to change their environment/habitat in their favor, producing a specific niche or creating a barrier to competing microbes^27^. In the rhizosphere, bacteria have also been described to alter the soil environment in such a way that certain microbial species are in advantage over others^28^. In our study, differential changes observed among cultivars upon *Pss* infection, may also reflect changes made by the pathogen *Pss* to the microhabitat. Probably, *Pss* could interact differently with the resident bacteria (pathogen-bacteria interaction) of each olive cultivar. Indeed, both cultivars had a distinct initial bacterial community, which after *Pss* interaction may probably lead to higher community fluctuation in cv. *Verdeal Transmontana* than in cv. *Cobrançosa*. Although the results presented here are in accordance with the accepted idea that host microbiome is a key for plant capability to overcome a pathogen attack^29,30^, this assumption still needs to be confirmed with further work.

Changes in bacterial diversity and composition between asymptomatic and OK-symptomatic twigs were greater for endophytes than for epiphytes, thus suggesting a greater sensitivity of endophytes to *Pss* infection. While no comparative studies are available considering the effect of a plant disease in host bacterial epiphytic and endophytic community composition, Gomes *et al*.^16^ obtained an opposite result with fungal communities, being epiphytes more affected by bacterial disease than endophytes. Furthermore, we have detected distinct changes on endophytic communities from both cultivars. While OK symptoms (knots) increased the diversity of endophytic bacterial in cv. *Cobrançosa*, the opposite was detected in cv. *Verdeal Transmontana*. Altogether, the results suggest that the interaction established between pathogen-host plant or pathogen-native bacterial community could benefit or inhibit specific bacterial endophytes. In fact, the capacity of *Pseudomonas* to affect the growth and density of other interacting bacteria has been described in different organisms, including plants^31–33^. This effect has been reported to be a consequence of the cooperation and competition of *Pseudomonas* species with other microorganisms^31,34^. Endophytically, *Pss* cells are organized in clusters, forming also biofilm layers^35^. The formation of this biofilm provides several advantages to certain bacteria, such as social cooperation, resource capture and protection from antimicrobials^36^, which may explain the increase of bacterial abundance in OK symptomatic twigs. Based on our results, such pathogen effect seems to have greater impact on the endophytic bacterial community when compared to epiphytic community.

In this study, a number of bacterial genera were found to be specifically associated with asymptomatic or symptomatic twigs of each cultivar. Among the genera most associated to OK-symptomatic twigs, both *Pantoea* and *Erwinia* have already been reported to occur in olive knots and suggested to be crucial for the development of OK disease^13^. Indeed, *Pantoea agglomerans* and *Erwinia toletana* have been frequently associated with olive knots^12–14,37^. Furthermore, when inoculated together with *Pss* they both promoted tumors size increases in olive trees^37,38^. Although it is not known how exactly *P. agglomerans* and *E. toletana* modulates the OK-disease severity, a number of studies provided evidences of a crosstalk between *Pss*, *P. agglomerans* and *E. toletana* that could have a role on *Pss* virulence^12,39^. Interestingly, apart from *Pss*, *Pantoea* and *Erwinia*, other *Pseudomonas* species were found to be associated with knots, both as an epiphyte and endophyte. A *Pseudomonas* spp. complex has been previously reported to be associated to plant diseases in different crops, such as *Solanum lycopersicum*^40^, *Prunus*^41^, citrus^42^ and mango^43^. In our study, the frequent occurrence of a *Pseudomonas* sp. with *Pss* in olive knots suggests that this consortium is stable and both organisms probably benefit from the presence of each other. Further work needs to be done to establish whether *Pseudomonas* microflora contributes to the OK disease caused by *Pss*.

Although a broader and complete view of bacterial communities could have been achieved by using culture-independent methods^44^, such as metabarcoding approaches using 16S rDNA barcode, we have decided to identify bacterial communities using culture-dependent approach for obtaining bacterial isolates that could be used in the future. Although more limited in depicting whole bacterial communities, results have clearly revealed that host genotype and habitat strongly influence plant pathobiome. Therefore, by understanding OK pathobiome and its relation with disease susceptibility, an additional purpose of this work was to obtain bacterial isolates that could be used for envisaging new biological control methods against olive diseases. The occurrence of bacteria specifically associated with asymptomatic twigs, particularly in the resistant cv. *Cobrançosa*, may give hints about their role in disease control. Among the genera associated to asymptomatic twigs (either as an epiphyte and endophyte), *Bacillus* have been identified as the most promising in improving plant growth and controlling plant diseases^45^. In fact, there are many studies indicating the ability of *Bacillus* spp. to inhibit microbial pathogen growth either in soil or in plant tissues^45–47^. In olive, a few number of species belonging to this genus were described to have a high antagonistic potential, not only against *Verticillium dahliae*^48,49^, but also against *Pss*^50,51^. *Bacillus* spp. isolated from olive leaves were tested against *Pss*, revealing promising results in both *in vitro* and *in planta* assays^50,51^. In the present study, other genera (*Alcaligenes*, *Brevundimonas*, *Curtobacterium* and *Arthrobacter*) were also strongly correlated with asymptomatic tissues. Although *Alcaligenes* include clinically relevant strains^52,53^, some studies have been reporting the bacteriostatic and fungistatic activity (biocontrol activity) of some members against an array of plant pathogens^54–56^. Species belonging to *Brevundimonas* were previously described to confer fitness advantages to host plants, being indicated as potential soil bioremediators^57^ and plant growth promotors^58^. However, members of this genus are frequently known as causing severe infections in humans^59,60^, compromising their use in the control of plant diseases. Members of *Curtobacterium* have been mainly described as plant pathogens^61^ and have been found as endophytes on some woody plant species, such as coffee^62^, orange and tangerine^63^. However, there are also reports showing the biocontrol potential of some members of this genus. For example, *C. flaccumfaciens* revealed to inhibit the phytopathogens *Xyllela fastidiosa*^18,64^ and *V. dahliae*^65^. *Arthrobacter* includes a large number of widespread species, in particular in soil, and with great importance in environmental and industrial applications^66–68^. Apart from nitrogen fixation^69^, members of *Arthrobacter* genus also revealed antagonism towards several plant pathogens and capacity to inhibit plant diseases^70,71^. The role of these bacterial genera associated to asymptomatic twigs, on the defense of olive trees against OK disease remains a topic for further study.

In summary, with this work we revealed that olive bacterial communities change with OK disease. This effect was most notorious within endophytes than within epiphytes and was dependent on host cultivar. Indeed, we observed a greater effect of OK disease on bacterial community assemblage associated with cv. *Verdeal Transmontana* (more susceptible) than with cv. *Cobrançosa* (more resistant). Overall, the composition of bacterial community in olive knots seems to result from complex interactions between host plant-*Pss*-native bacteria. Our work also identified key bacterial genera (especially *Bacillus* and *Brevundimonas*) that could play an important role in the susceptibility/resistance of cultivars to OK disease. Understanding the mechanisms of interaction (cooperation *vs*. competition) and communication of these bacteria with *Pss* will shed light on the role of these bacteria on the process of OK disease development.

## Material and Methods

### Asymptomatic and diseased twigs sampling

Sample collection was performed during spring 2015, in two olive orchards located in Mirandela (northeast of Portugal), at coordinates N41°32.593′; W07°07.445′ (orchard 1) and N41°32.756′; W07°07.590′ (orchard 2). These orchards contain two olive cultivars of varying susceptibilities to OK disease (*i.e*., cv. *Verdeal Transmontana* is more susceptible than cv. *Cobrançosa*^16^), growing together within 7 m of each other, under identical environmental conditions and management practices (integrated production guidelines). In each orchard, seven olive trees of each cultivar were randomly selected. Both asymptomatic and OK-symptomatic twigs (with knots) were collected from the same branch, at mid-canopy height, using sterilized shears and gloves. The collected samples were individually placed into sterile roll bags, brought to the lab on ice, and then stored at 4 °C until bacterial isolation, which was performed within one week.

### Epiphytic and endophytic bacterial isolation

After removing the leaves from twigs, the epiphytic bacteria were isolated from pieces of five asymptomatic twigs or knots (with ca. 1-gram weight) cut from symptomatic twigs. These plant segments were individually immersed in 9 mL peptone water (10 g/L peptone, 5 g/L sodium chloride) and shaken for one hour, at 100 rpm at room temperature. Aliquots of 1 ml of the bacterial suspension were then incorporated in triplicate onto 10 mL of Luria Bertani (LB) agar medium (10 g/L peptone, 5 g/L yeast extract, 5 g/L sodium chloride, 10 g/L agar) and incubated at 25 °C, in the dark until bacterial growth. Daily observations were performed in order to isolate and count bacterial colonies (CFU, Colony Forming Units). For isolation, single colonies were picked up, cultured in sterile LB plates and stored at 4 °C when full growth was observed. The abundance of epiphytes was expressed as log CFU/cm^2^, representing the number of colonies per cm^2^ of twig/knot surface. Surface of asymptomatic twigs and knots were measured based on cylinder (A = 2πrh + 2πr^2^) and sphere (A = 4πr^2^) area equations, respectively, where A is the area, r is the radius and h is the height of plant segments. The average twig and knot segments area were 11.0 ± 3.6 and 2.9 ± 1.3 cm^2^, respectively, for cv. *Cobrançosa*, and 11.0 ± 2.3 and 2.9 ± 1.2 cm^2^, respectively, for cv. *Verdeal Transmontana*. A total of 280 plant segments (2 olive orchards × 2 olive cultivars × 7 olive trees × 5 twigs or 5 knots) were used for isolating epiphytes.

Endophytes were isolated from the same twig/knot segments used to isolate epiphytes. For this, plant segments were surface sterilized by immersion in ethanol 70% (v/v) for 1 min, followed by sodium hypochlorite 3% (v/v) for 1 min, and then rinsed three times in sterile distilled water (1 min, each). After drying, each twig/knot was cut into segments (ca. 4–5 mm). Five sterilized segments per twig/knot were aseptically transferred onto LB medium, in quintuplicate, and incubated at 25 °C in the dark, until bacterial growth. Cultures were daily monitored and single colonies emerging from tissues segments were counted and subcultured into LB medium in order to obtain pure cultures. Endophytes were isolated from the same 280 plant segments used for epiphytes isolation, corresponding to a total of 7,000 inoculated plant pieces (280 plant segments × 25 pieces *per* segment).

### DNA isolation and 16S rDNA sequencing

The taxonomic identification of bacterial isolates was performed by using both morphological and molecular approaches. Bacterial isolates were firstly grouped based on the cultural features of their colonies, such as colony color, size, shape, opacity, elevation, and margin surface. From each morphotype, two isolates were selected for molecular identification, raising a total of 294 bacteria isolates from twigs and tumors of both *Cobrançosa* (78 and 66 isolates, respectively) and *Verdeal Transmontana* (92 and 58 isolates, respectively) cultivars. Bacterial DNA was extracted using *REDExtract-N-Amp™ Plant PCR* kit (Sigma, Poole, UK), following manufacturer instructions, and used for PCR amplification of V1–V4 regions from 16S rRNA. For PCR reaction, 3 µL of extracted DNA was used in a 50 µL reaction mixture, containing 0.25 µL of each dNTP at 10 mM, 7 µL of 10x buffer, 2.5 µL of 25 mM MgCl_2_, 0.25 µL of DFS-*Taq* DNA Polymerase (5 units/µL) and 1 µl of each primer at 10 µM (V1F: 5′- AGAGTTTGATCCTGGCTCAG-3′; V4R: 5′-TACNVGGGTATCTAATCC-3′)^72^. Amplifications occurred in a MyCycler™ Thermocycler (Bio-Rad), using the following PCR program: 94 °C for 5 min, followed by 35 cycles of 94 °C for 50 sec, 45 °C for 30 sec, 72 °C for 90 sec, with a final extension of 72 °C for 5 min. PCR product was sequenced by Macrogen Inc. (Madrid) and taxonomic identification was performed by using the NCBI database (http://www.ncbi.nlm.nih.gov) and BLAST algorithm. Operational taxonomic units (OTUs) displaying the lowest E-value and the highest identity score were identified to bacterial species (when identity presented a value >98%) or genus (when presenting 95% to 97% identity). For sequence identities <95%, OTUs were labelled as ‘unknown’. Identified bacterial isolates were preserved in the culture collection of the Mountain Research Centre (CIMO), Instituto Politécnico de Bragança.

### Data analysis

The cultivable epiphytic or endophytic bacterial community of asymptomatic and symptomatic (*i.e*., knots) twigs, present in both cvs. *Cobrançosa* and *Verdeal Transmontana*, were compared for determining if their variation is affected by plant host and plant habitat. *Pss* abundance was always excluded from analyses, for determining the true bacterial community changes and not be affected by the overabundance of the pathogen *Pss* in the symptomatic twigs (knots).

#### Bacterial diversity analyses

Diversity of bacterial communities was assessed by evaluating the abundance (relative number of isolates *per* tree), richness (number of OTUs *per* tree) and Shannon-Wiener Index (*H*’), determined by the *R* software^73^ and using an OTUs abundance matrix. The percentual changes of these diversity parameters occurring on OK-symptomatic twigs in relation to asymptomatic twigs was calculated by using the following formula: percentual changes (%) = [(symptomatic twigs − asymptomatic twigs)/symptomatic twigs] × 100. Estimated changes are presented as the mean of replicates (*i.e*., tree = 14, corresponding to 2 olive orchards × 7 trees) and respective SE values). Differences among means were determined by an analysis of variance (ANOVA) with *R* software, where the means were compared using Tukey’s test (*p* < 0.05).

#### Comparison of bacterial communities composition

Multivariate statistical analyses were performed to describe differences on the bacterial communities’ composition, among twig status (asymptomatic *vs*. OK-symptomatic) and plant habitat (epiphytic *vs*. endophytic). All statistical analyses were performed using the *R* software^73^. Non-metric multidimensional scaling (NMDS) was performed using Bray-Curtis index obtained from a normalized abundance OTU matrix, in order to calculate the dissimilarity in the composition of bacterial communities. Kruskal’s stress was used to estimate the model’s goodness of fit (commonly acceptable when lower than 0.2)^74^. A one-way analysis of similarity (ANOSIM) was also performed using Bray–Curtis distance matrices, for finding significant differences between the bacterial community groups observed in NMDS ordination. This analysis generates a *R*-value, which range from 0 (completely similar) to 1 (completely different), associated to a *p*-value (significant when lower than 0.05)^75^. Both NMDS and ANOSIM analyses were performed using the “vegan”^76^ package (*metaMDS* and *anosim* functions, respectively). For determining the main taxonomic changes that occur in the bacterial communities among samples, the mean percentage of bacterial abundance at different taxonomic levels (phylum, family and genus) was calculated across asymptomatic and OK-symptomatic (knots) twigs. Differences among means were determined by ANOVA with *R* software^73^, and means were compared using the Tukey’s test (*p *< 0.05). A log_10_ transformation of bacterial abundance was used for meeting ANOVA assumptions.

#### Factors driving the shaping of bacterial communities

In order to assess the contribution of distinct factors [host cultivar (cv. *Cobrançosa vs*. cv. *Verdeal Transmontana*), disease (asymptomatic *vs*. OK-symptomatic) and plant habitat (epiphytic *vs*. endophytic)] to differences detected on bacterial communities, a variation partitioning analysis was performed. For this, the *varpart* function included in the “vegan” package of *R* software^73^ and a normalized abundance OTU matrix were used. The significance of each fraction was tested using the *anova.cca* function.

#### Identification of bacterial consortium associated to each host cultivar and twig status

A multiple factor analysis (MFA) was used to identify bacterial genera associated to a specific host cultivar and disease symptoms. For this analysis, only the epiphytic and endophytic bacterial genera with the greatest power to separate asymptomatic from OK-symptomatic and cv. *Cobrançosa* from cv. *Verdeal Transmontana* twigs were used. These bacterial genera were identified by using a random forest analysis, which was computed with the “RandomForest” package^17^ from *R*. The importance of bacterial genera for distinguishing communities was measured by considering the decrease in mean Gini coefficient, where a higher decrease will imply a higher importance^77^. MFA was computed by using the “FactoMineR”^78^ package from *R*. Bacterial genera and variables were graphically represented by the first two dimensions. Then, *Spearman* correlations were performed through the “corrplot”^79^ package from *R*, to check the correlation of pre-selected epiphytic and endophytic bacterial genera with the relative abundance of *Pss*.

## Supplementary information

Supplementary information.
