# Supplementary material for: Impact of plant genotype and plant habitat in shaping bacterial pathobiome: a comparative study in olive tree
Source: Sci Rep. 2020 Feb 26;10:3475. doi: 10.1038/s41598-020-60596-0 (PMC7044170; doi:10.1038/s41598-020-60596-0)
Supplement: Supplementary file 1 — Supplementary information. [file 41598_2020_60596_MOESM1_ESM.docx]

**Supplementary Information**

**Article title:** Impact of plant genotype and plant habitat in shaping bacterial pathobiome: a comparative study in olive tree

**Authors:** Diogo Mina, José Alberto Pereira, Teresa Lino-Neto and Paula Baptista

**The following Supplementary Information is available for this article:**

**Fig. S1 -** Relative abundance (%) of the global epiphytic and endophytic bacteria communities isolated from twigs of olive tree. Bacterial communities are discriminated at genus, family and phylum levels.

**Fig. S2** - Changes (%) on epiphytic and endophytic bacterial abundance, richness and diversity, occurring on asymptomatic and OK-symptomatic twigs of olive trees from cvs. *Cobrançosa* and *Verdeal Transmontana*. Boxplots depict medians (central horizontal lines), the inter-quartile ranges (boxes), 95% confidence intervals (whiskers), and outliers (dots). Statistically differences between pairs of values are showed over horizontal lines. Abbreviation: n.s., not significant.

**Fig. S3** - Ranking of relative importance of each bacterial genus for distinguishing asymptomatic from OK-symptomatic twigs of both cvs. *Cobrançosa* and *Verdeal Transmontana*, considering epiphytic and endophytic bacterial communities. Mean Decrease Gini value measure the importance of bacterial genera, with highest values representing the best predictors. Genera in bold were considered as the most relevant to distinguish the presence of disease symptoms on twigs.

**Fig. S4** – Ranking of relative importance of each bacterial genus for distinguishing cv. *Cobrançosa* from cv. *Verdeal Transmontana* on asymptomatic and OK-symptomatic twigs, considering epiphytic and endophytic bacterial communities. Mean Decrease Gini value measure the importance of bacterial genera, with highest values representing the best predictors. Genera in bold were considered as the most relevant to distinguish host cultivars.

**Table S1** - Epiphytic and endophytic relative abundance of *Pseudomonas savastanoi* pv. *savastanoi* detected on asymptomatic and OK-symptomatic twigs from both cultivars (*Cobrançosa* and *Verdeal Transmontana*). Values are presented as means ± SE. Different superscript letters denote statistically significant differences (*p*<0.05) among twigs with/without OK-symptoms within each epiphytic/endophytic habitats.

**Table S2** - Total variance (*varpart*) explained by host cultivar (*Cobrançosa vs. Verdeal Transmontana*), twigs with/without OK-symptoms (asymptomatic *vs*. OK-symptomatic) and plant habitat (epiphytic *vs*. endophytic) on the bacterial community composition. ANOVA analysis was performed to test the significant differences (*p*-value).

**Table S3** - *Spearman* correlation analysis between bacterial genera abundances and *Pseudomonas savastanoi* pv. *savastanoi* abundance within epiphytic and endophytic communities of each olive tree cultivar (*Cobrançosa* and *Verdeal Transmontana*). Only significant correlation values are presented.


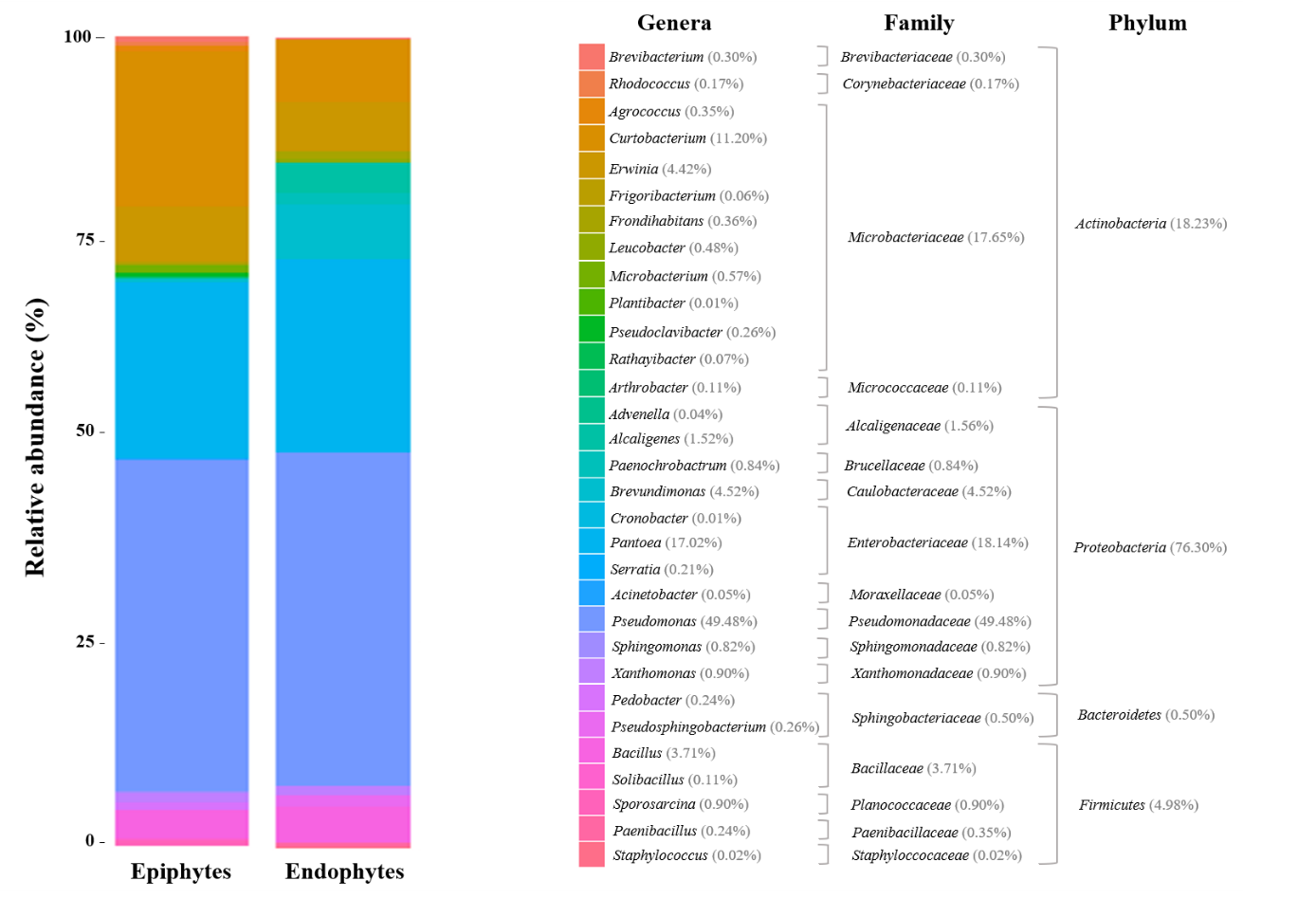


**Fig. S1** - Relative abundance (%) of the global epiphytic and endophytic bacteria communities isolated from twigs of olive tree. Bacterial communities are discriminated at genus, family and phylum levels.


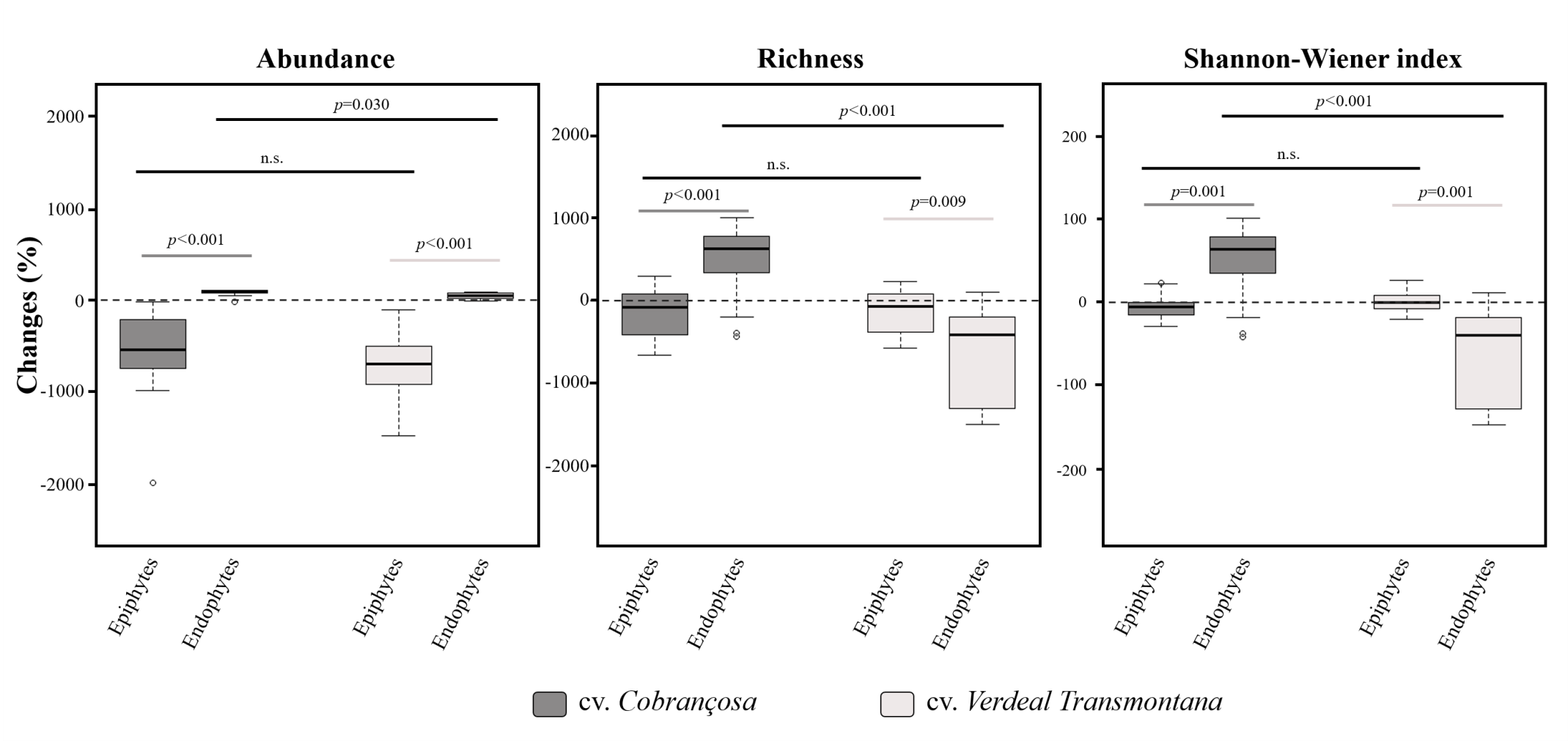


**Fig. S2 -** Changes (%) on epiphytic and endophytic bacterial abundance, richness and diversity, occurring on asymptomatic and OK-symptomatic twigs of olive trees from cvs. *Cobrançosa* and *Verdeal Transmontana*. Boxplots depict medians (central horizontal lines), the inter-quartile ranges (boxes), 95% confidence intervals (whiskers), and outliers (dots). Statistically differences between pairs of values are showed over horizontal lines. Abbreviation: n.s., not significant.


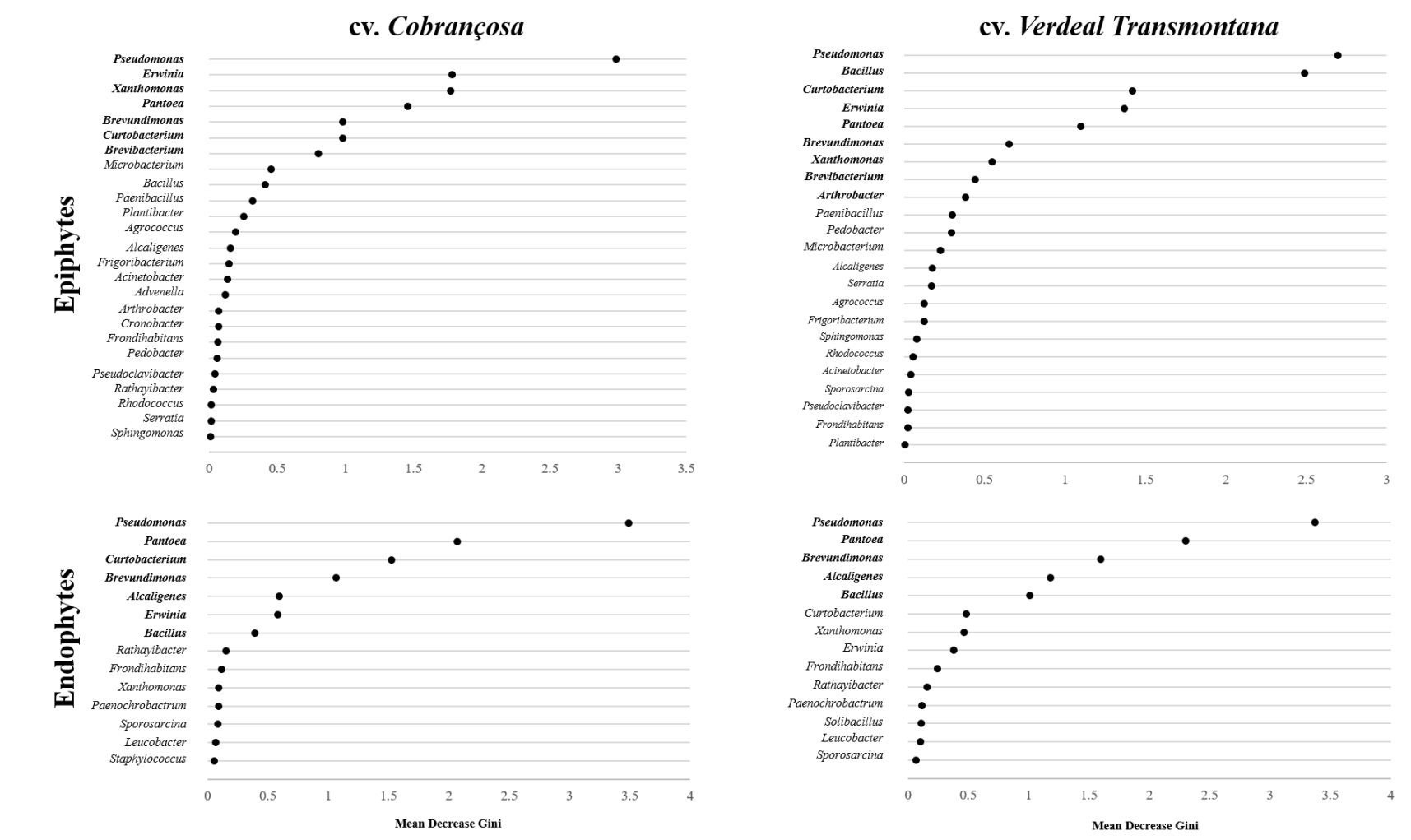


**Fig. S3** - Ranking of relative importance of each bacterial genus for distinguishing asymptomatic from OK-symptomatic twigs of both cvs. *Cobrançosa* and *Verdeal Transmontana*, considering epiphytic and endophytic bacterial communities. Mean Decrease Gini value measure the importance of bacterial genera, with highest values representing the best predictors. Genera in bold were considered as the most relevant to distinguish the presence of disease symptoms on twigs.


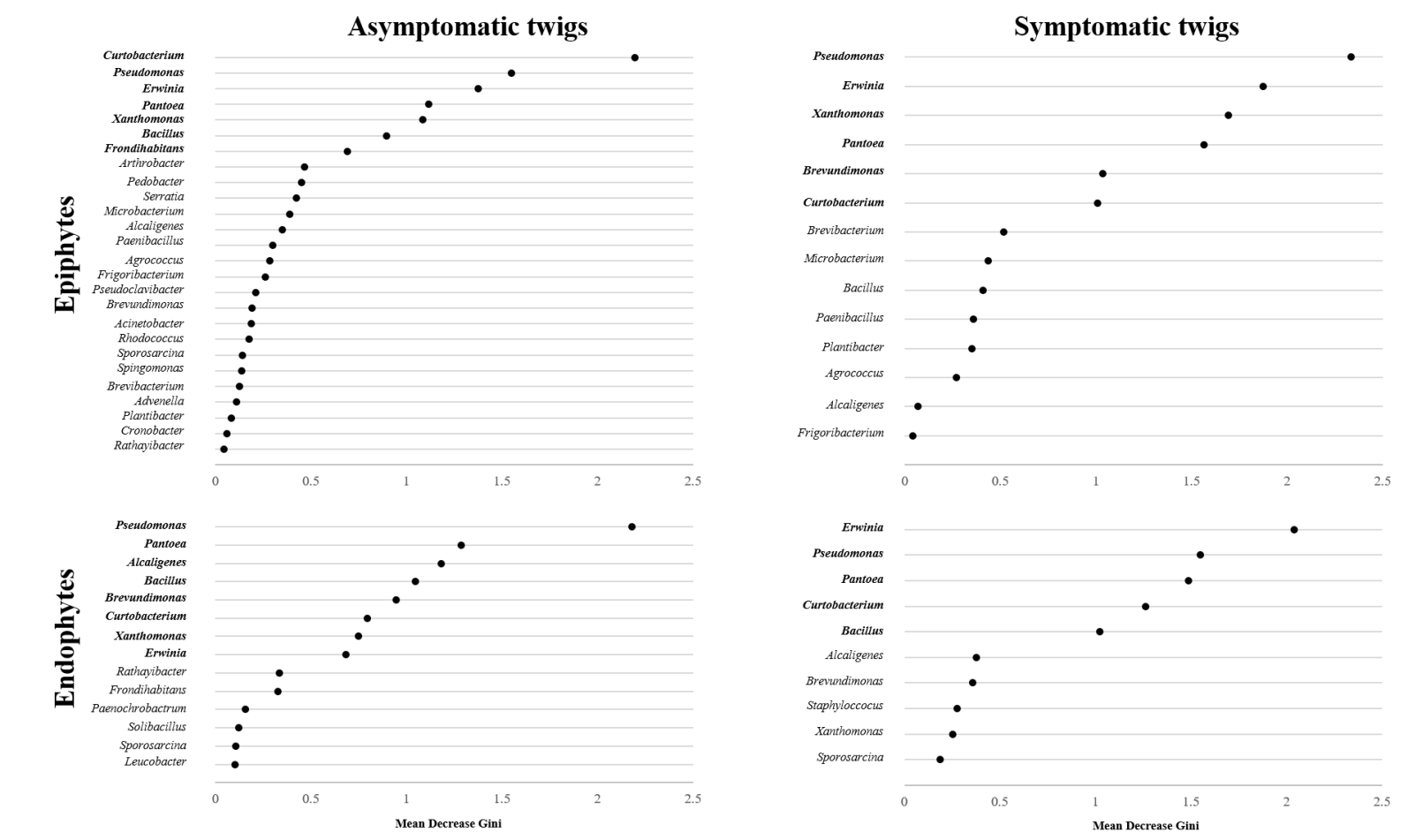


**Fig. S4** – Ranking of relative importance of each bacterial genus for distinguishing cv. *Cobrançosa* from cv. *Verdeal Transmontana* on asymptomatic and OK-symptomatic twigs, considering epiphytic and endophytic bacterial communities. Mean Decrease Gini value measure the importance of bacterial genera, with highest values representing the best predictors. Genera in bold were considered as the most relevant to distinguish host cultivars.

**Table S1** – Epiphytic and endophytic relative abundance of *Pseudomonas savastanoi* pv. *savastanoi* detected on asymptomatic and OK-symptomatic twigs from both cultivars (*Cobrançosa* and *Verdeal Transmontana*). Values are presented as means ± SE. Different superscript letters denote statistically significant differences (*p*<0.05) among twigs with/without OK-symptoms within each epiphytic/endophytic habitats.

| **Cultivar** | **Occurrence of OK symptoms** | **Epiphytic** | **Endophytic** |
| --- | --- | --- | --- |
|  |  |  |  |
| **cv. *Cobrançosa*** | **Asymptomatic** | 11.4±2.3^c^ | 6.7±2.6^c^ |
|  | **Symptomatic** | 34.8±2.2^a^ | 15.7±0.8^bc^ |
|  | **Asymptomatic+symptomatic** | 23.1±2.7^b^ | 14.8±3.5^c^ |
|  | | | |
| **cv. *Verdeal Transmontana*** | **Asymptomatic** | 12.3±0.5^c^ | 13.5±2.1^bc^ |
|  | **Symptomatic** | 12.7±2.8^bc^ | 26.9±1.2^a^ |
|  | **Asymptomatic+symptomatic** | 12.5±1.4^bc^ | 20.2±1.7^ab^ |
|  | | | |
| **Total** | **Asymptomatic** | 12.1±1.8^cd^ | 10.1±1.8^d^ |
|  | **Symptomatic** | 23.6±2.4^a^ | 21.3±1.3^ab^ |
|  | **Asymptomatic+symptomatic** | 17.8±1.7^abc^ | 15.7±1.3^bcd^ |
|  | | | |

**Table S2** - Total variance (*varpart*) explained by host cultivar *(Cobrançosa vs. Verdeal Transmontana*), twigs with/without OK-symptoms (asymptomatic *vs*. OK-symptomatic) and plant habitat (epiphytic *vs.* endophytic) on the bacterial community composition. ANOVA analysis was performed to test the significant differences (*p*-value).

| **Effect** | **Target** | ***Varpart* (%)** | ***p-*value** |
| --- | --- | --- | --- |
|  |  |  |  |
| **Occurrence of**  **OK symptoms**  (Asymptomatic *vs*.  OK- symptomatic) | Total community | 7.3% | 0.005 |
|  |  |  |  |
|  | cv. *Cobrançosa* community | 8.4% | 0.005 |
|  | cv. *Verdeal* community | 20.5% | 0.005 |
|  |  |  |  |
|  | Epiphytic community | 4.7% | 0.005 |
|  | Endophytic community | 11.7% | 0.005 |
|  |  |  |  |
| **Plant habitat**  (Epiphytic *vs*. Endophytic) | Total community | 7.1% | 0.005 |
|  |  |  |  |
|  | cv. *Cobrançosa* community | 11.4% | 0.005 |
|  | cv. *Verdeal* community | 13.8% | 0.005 |
|  |  |  |  |
|  | Symptomatic community | 26.8% | 0.005 |
|  | Asymptomatic community | 7.5% | 0.005 |
|  |  |  |  |
| **Host cultivar**  (*Cobrançosa* vs.  *Verdeal Transmontana*) | Total community | 3.6% | 0.005 |
|  |  |  |  |
|  | Symptomatic community | 24.4% | 0.005 |
|  | Asymptomatic community | 2.5% | 0.005 |
|  |  |  |  |
|  | Epiphytic community | 14.7% | 0.005 |
|  | Endophytic community | 10.6% | 0.005 |
|  |  |  |  |

**Table S3** - *Spearman* correlation analysis between bacterial genera abundances and *Pseudomonas savastanoi* pv. *savastanoi* abundance within epiphytic and endophytic communities of each olive tree cultivar (*Cobrançosa* and *Verdeal Transmontana*). Only significant correlation values are presented.

| **Plant habitat** | **Plant cultivar** | **Genera** | **Correlation coefficient** | ***p-*value** |
| --- | --- | --- | --- | --- |
|  |  |  |  |  |
| Epiphytic communities | cv. *Cobrançosa* | *Pseudomonas*  *Erwinia* | 0.629  0.708 | 0.016  0.005 |
|  |  |  |  |  |
|  | cv. *Verdeal Transmontana* | *Pantoea*  *Pseudomonas* | 0.622  0.570 | 0.018  0.033 |
|  |  |  |  |  |
| Endophytic communities | cv. *Cobrançosa* | *Bacillus*  *Curtobacterium* | -0.663  -0.529 | 0.009  0.049 |
|  |  |  |  |  |
|  | cv. *Verdeal Transmontana* | *Pseudomonas*  *Brevundimonas* | 0.674  -0.566 | 0.008  0.035 |
|  |  |  |  |  |
